# Case Report: Complete Maternal Uniparental Isodisomy of Chromosome 5 (iUPD(5)mat) With *PCSK1* Nonsense Variant in an Infant With Recurrent Diarrhea

**DOI:** 10.3389/fgene.2021.668326

**Published:** 2021-05-06

**Authors:** Yanyan Qian, Bingbing Wu, Renchao Liu, Yulan Lu, Ping Zhang, Caihong Shao, Ying Huang, Huijun Wang

**Affiliations:** ^1^Center for Molecular Medicine, National Children's Medical Center, Pediatrics Research Institute, Children's Hospital of Fudan University, Shanghai, China; ^2^Department of Gastroenterology, Pediatric Inflammatory Bowel Disease Research Center, National Children's Medical Center, Children's Hospital of Fudan University, Shanghai, China

**Keywords:** diarrhea, *PCSK1* gene, uniparental isodisomy, infant, genetics

## Abstract

Congenital diarrhea diseases are a heterogeneous group of conditions and are the major cause of neonatal mortality worldwide. Proprotein convertase 1/3 (PC1/3) deficiency has been associated with severe malabsorptive diarrhea, obesity, and certain endocrine abnormalities. We report an infant born to non-consanguineous parents who is diagnosed with PC1/3 deficiency due to nonsense homozygous variant (c.238 C>T, p.Arg80Ter) in the *PCSK1* gene, identified by Trio-exome sequencing (Trio-ES). The baby girl presented with recurrent diarrhea, transient liver dysfunction and hypoglycemia. Trio-ES showed complete maternal uniparental isodisomy (iUPD) of chromosome 5. Our finding provides accurate genetic counseling to this family and expands the clinical spectrum of iUPD with pathogenic variants causing recessive disease.

## Background

Congenital diarrhea diseases (CDDs) are a heterogeneous group of conditions characterized by watery diarrhea and are the major cause of neonatal mortalities worldwide (Younis et al., 2020). The World Health Organization defines diarrhea as three or more loose or liquid stools per day or an increase in defecation frequency. Early diagnosis of CDD is critical to prevent the progression of the disease to avoid adverse outcomes. With genetic testing increasingly applied to clinical use, the genetic causes of CDDs were gradually revealed and the candidate of causative genes was accumulating (Elkadri, 2020; Younis et al., 2020). These genes were functionally divided into five different subgroups, including the defects in epithelial nutrient and electrolyte transport, epithelial enzyme and metabolism, epithelial trafficking and polarity, enteroendocrine cell, and immune system (Thiagarajah et al., 2018; Elkadri, 2020).

Currently, four genes are reported to be related to enteroendocrine cell dysfunction, including *NEUROGS* (causing congenital malabsorptive diarrhea 4), *PCSK1* (causing obesity with impaired prohormone processing/proprotein convertase 1/3 deficiency, or PCSK1 deficiency) (Younis et al., 2020), and genes causing a syndromic disease with diarrhea—*ARX* (leading to X-linked lissencephaly 2 or hydranencephaly with abnormal genitalia) and *RFX* (leading to Mitchell-Riley syndrome) (Thiagarajah et al., 2018; Elkadri, 2020). Among the above disorders, PCSK1 deficiency is an age-related CDD, with neonatal-onset severe malabsorptive diarrhea, but the intestinal phenotype will disappear later and the patient would even develop obesity with multiple endocrine abnormalities (Jackson et al., 1997; Frank et al., 2013; Pepin et al., 2019).

Uniparental isodisomy (iUPD), which means both alleles originating from a single-parental homologous chromosome, is an underestimated cause of autosomal recessive disorders (Yamazawa et al., 2010). Currently, nine patients had been reported with uniparental disomy of chromosome 5 [UPD(5)] with homozygous variants (Park et al., 2019; Gonzalez-Quintana et al., 2020). However, maternal iUPD of chromosome 5 (iUPD(5) mat) with a homozygous pathogenic variant in *PCSK1* has never been reported.

Here, we report the first case of iUPD(5)mat leading to a homozygous nonsense variant in *PCSK1* identified by Trio-ES. Clinicians and genetic counselors should be aware that for offspring of non-consanguineous parents, autosomal recessive disorders due to homozygous variants can occur as a result of uniparental isodisomy.

## Case Presentation

**Figure 1:**
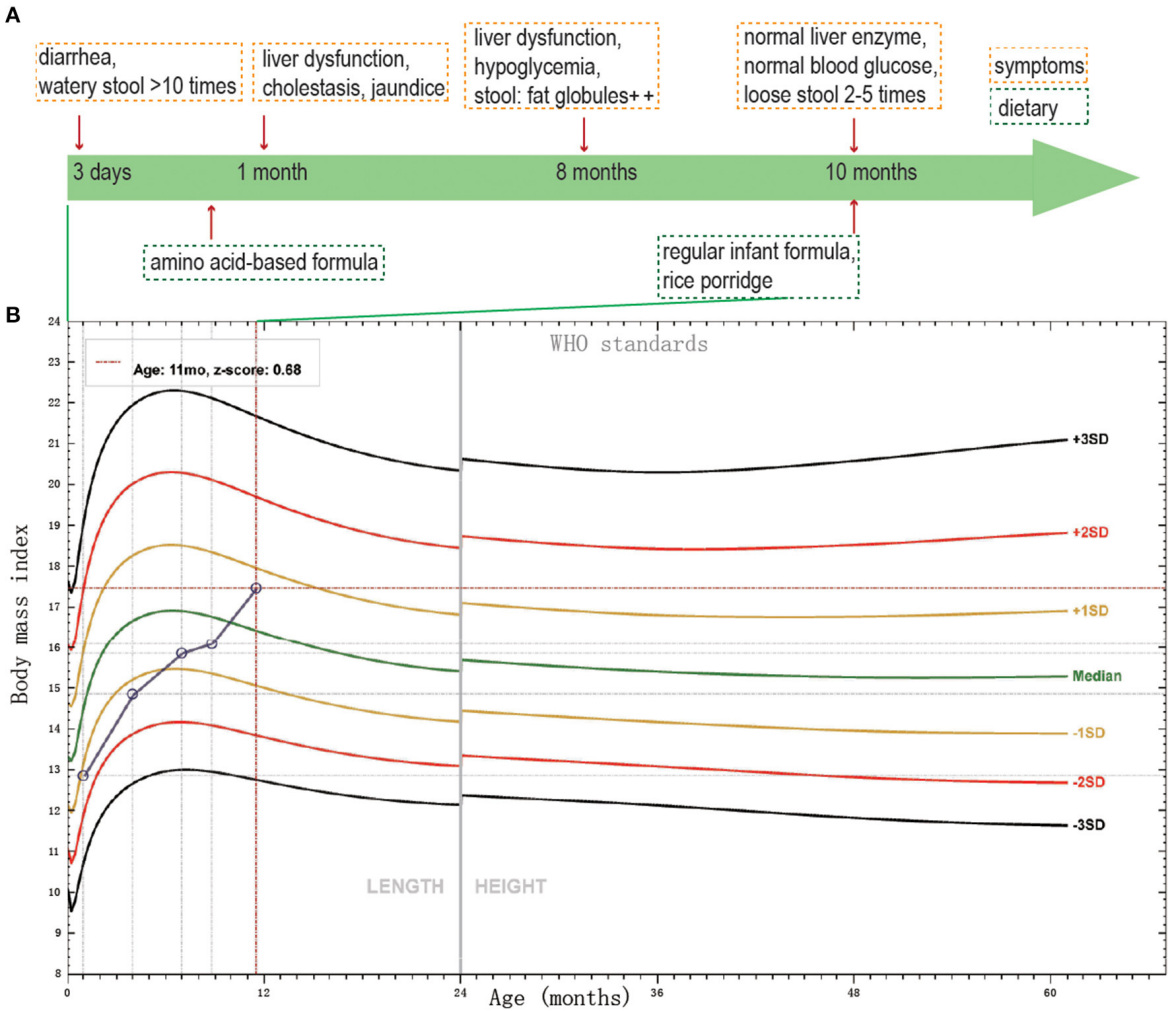
The thriving records of clinical features of the patient. **(A)** The clinical symptoms and dietary of the patient. **(B)** BMI SD score of the proband, according to WHO standards, from birth to age 11 months.

The proband was a 7 month-23-day old girl at admission. She was born to a healthy non-consanguineous couple after an uneventful full-term pregnancy and was born without anomalies. But on day 3, the infant began diarrhea with watery stool over ten times a day. When she was 1 month old, she manifested jaundice and cholestasis with mild abnormal liver enzymes. During the first month, different types of infant formulas and breast milk were performed on her, but the diarrhea was not controlled. Then she was fed with amino acid-based formula until 8 months old, with diarrhea persisting. At her 8 months old, the routine stool test of her was not very informative, except for some fat globules existing. The enteric adenovirus, rotavirus, and norovirus were all negative tested by the immune gold standard method. The immunoglobulin, including IgA, IgG, IgM and IgE were normal, meaning that she had no immunity system deficiency. Her liver enzymes were still abnormal, AST 85.6 (normal 13–35 U/L); ALT 129.2 (normal 7–40 U/L), together with asymptomatic hypoglycemia, blood glucose 3.8 (normal 3.9–6.1 mmol/L). However, when she was 10 months old, her liver enzyme and blood glucose turned to normal. From then on, rice porridge was added to her meals with regular infant formula, and she can drink 300–500 ml water and 1,000 ml milk per day. Now she had loose stool twice to five times per day. The thriving records were shown in Figure 1A.

She got the physical examination five times. The height and weight were 50 cm and 3.3 kg at birth, 60.2 cm and 5.5 kg at 3 months old, 69 cm and 7.7 kg at 6 months old, 72 cm and 8.5 kg at 8 months old, 75 cm and 10 kg at 11 months old. The height and body mass index (BMI) score of the proband from birth to age 11 months was shown in Figure 1B. The BMI score increased gradually during the past 11 months. Her mother and father's height, BMI were 160 cm, 25.4 kg/m^2^ and 175 cm, 37.5 kg/m^2^, respectively. Pretest counseling was performed by the physician. Informed consent was signed by the parents. The criteria of genetic testing received approval from the ethics committees of the Children's Hospital, Fudan University (2015–130).

Molecular genetic analysis was ordered when the girl was 7 month-23-day old. Genomic DNA was extracted from the infant's and her parents' peripheral blood using a whole blood genomic DNA extraction kit (Qiagen, German). DNA fragments were enriched using the Agilent SureSelect XT Human All Exon 50 Mb kit (Santa Clara, CA). Then DNA libraries were sequenced on the HiSeq2000/2500 sequencer according to the manufacturer's instructions (Illumina, San Diego, CA). The data analysis method followed the pipeline established in-house (Yang et al., 2019). The criteria of the molecular diagnosis followed the American College of Medical Genetics (ACMG) guidelines. We classified the variant to be a pathogenic variant, according to PVS1+PM2+PP4. UPD was detected using “B Allele Frequency” (BAF) (van Riet et al., 2018). The distribution of variant heterozygosity on each chromosome was calculated to scan the UPD event. The nonsense variant of *PCSK1* was confirmed by Sanger sequencing using ABI 3,730 Genetic Analyzer (Applied Biosystems). Agilent SurePrint G3 comparative genomic hybridization (CGH) and SNP 4 × 180K microarray (Agilent Technologies, USA) was used to confirm the UPD(5) following the manufacturer's instructions. We used Agilent Cytogenomics software package for CNVs and the absence of heterozygosity (AOH) calling and visualization.

**Figure 2:**
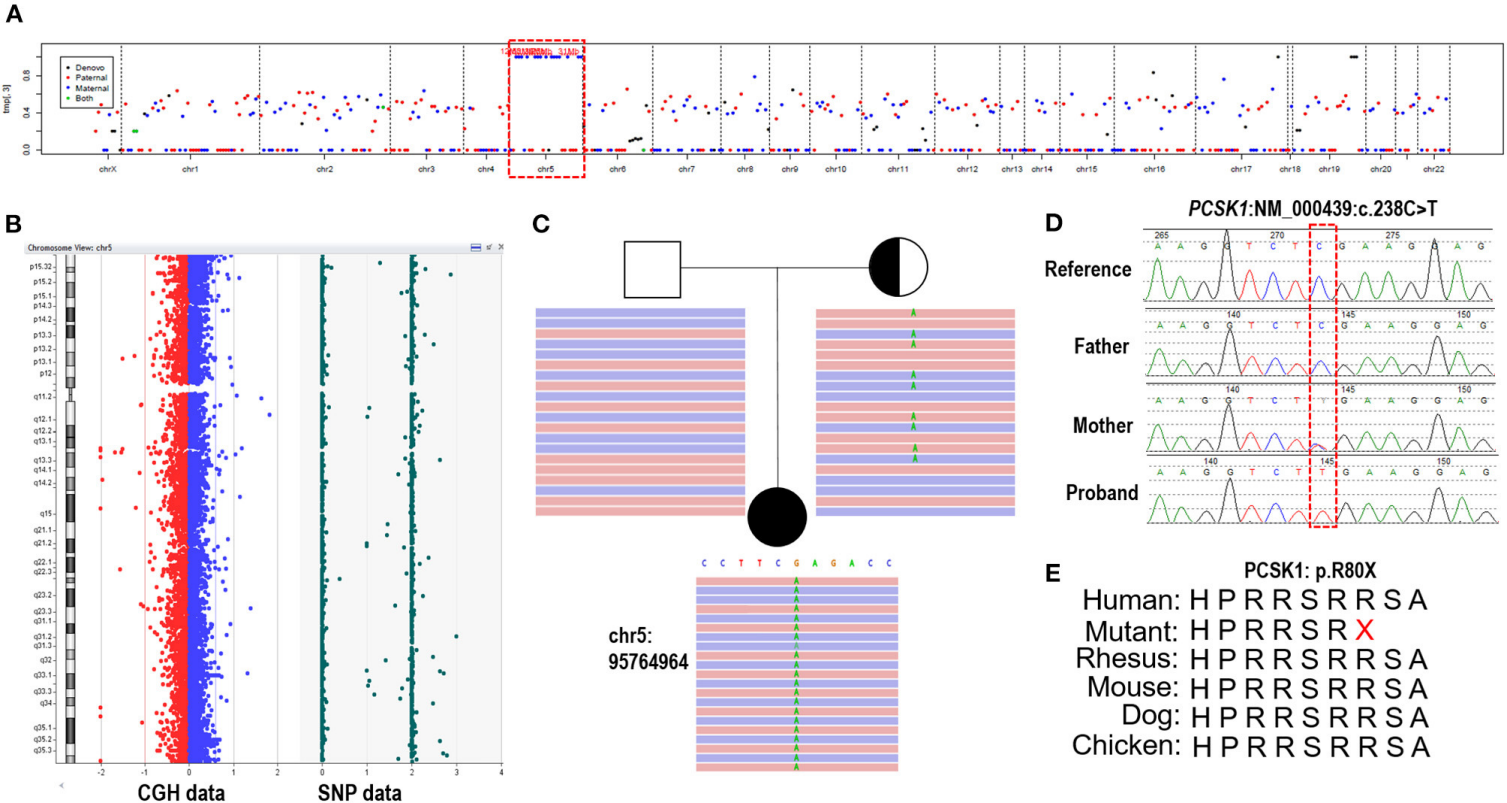
The genetic test results of the patient. **(A)** Trio-ES identified homozygous variants on chromosome 5 and inherited from her mother, suggesting that the proband has UPD(5)mat. **(B)** Array-CGH confirmed UPD(5) by showing the copy neutral AOH across the whole chromosome 5. **(C)** A homozygous nonsense variant on *PCSK1* (c.238C>T) is detected in the proband. Her mother is heterozygous and her father is normal. **(D)** The variant is confirmed by Sanger sequencing. **(E)** The variant site is conserved in different species.

Trio-ES data showed that 97.9% (1168/1193) variants had a BAF higher than 0.95 on chromosome 5, meaning the absence of heterozygosity (AOH), which is inherited from the mother (Figure 2A). Array-CGH showed no copy number variants in the proband but complete UPD(5) by showing AOH across the entire chromosome (Figure 2B). The sizes of AOH in this patient was 172.48 Mb detected by array-CGH, and 170.95 Mb detected by Trio-ES, respectively. Meanwhile, a homozygous nonsense variant in *PCSK1* (NM_000439: c.238C>T, p.Arg80Ter) was detected in the proband, while her mother was a heterozygous carrier and her father was normal (Figure 2C). This variant was confirmed by Sanger sequencing (Figure 2D). The variant whose site is highly conserved among different species caused a protein truncation (Figure 2E). Trio-ES did not identify any other pathogenic or likely pathogenic variants associated with the patient's clinical features or other inherited diseases.

Combining the clinical phenotype (continuous neonatal diarrhea, BMI increase) and the molecular genetic finding, the proband was diagnosed with congenital proprotein convertase 1/3 deficiency caused by a *PCSK1* homozygous pathogenic variant due to iUPD(5)mat.

## Discussion

*PCSK1* encodes the proprotein convertase subtilisin/kexin type 1, which involves the proteolytic activation of polypeptide hormones and neuropeptides precursors. Defects in this gene have been associated with susceptibility to obesity (Ramos-Molina et al., 2016) and congenital proprotein convertase 1/3 deficiency (PC1/3 deficiency) (Martin et al., 2013; Pepin et al., 2019)—also called obesity with impaired prohormone processing [OMIM: 600955]. Here, we report a new case that suffered from recurrent diarrhea since 3 days old, with a homozygous nonsense pathogenic variant in *PCSK1* (Arg80Ter). The variant was predicted to lead to a premature stop codon. The truncated protein may lose the entire catalytic domain, P domain, C-terminal tail, and part propeptide. This variant was once detected in a family with obesity disorder. The variant was overexpressed in the 293T cell line, and they found that the activity of wild-type PC1/3 was inhibited (Philippe et al., 2015). However, that is an experiment not with tissue/cells from the patient. In addition, truncating protein could be generated when some mRNA escapes nonsense-mediated decay (NMD). Therefore, the detection of truncating protein in a cell line does not rule out the possibility of NMD. Further studies will be needed to determine whether NMD occurs for this variant. Besides, all of the heterozygous carriers in the family showed obesity or overweight; the lowest BMI of the carrier was 26.91. The patient's mother in our study show overweight (BMI: 25.4) but not obesity.

**Figure 3:**
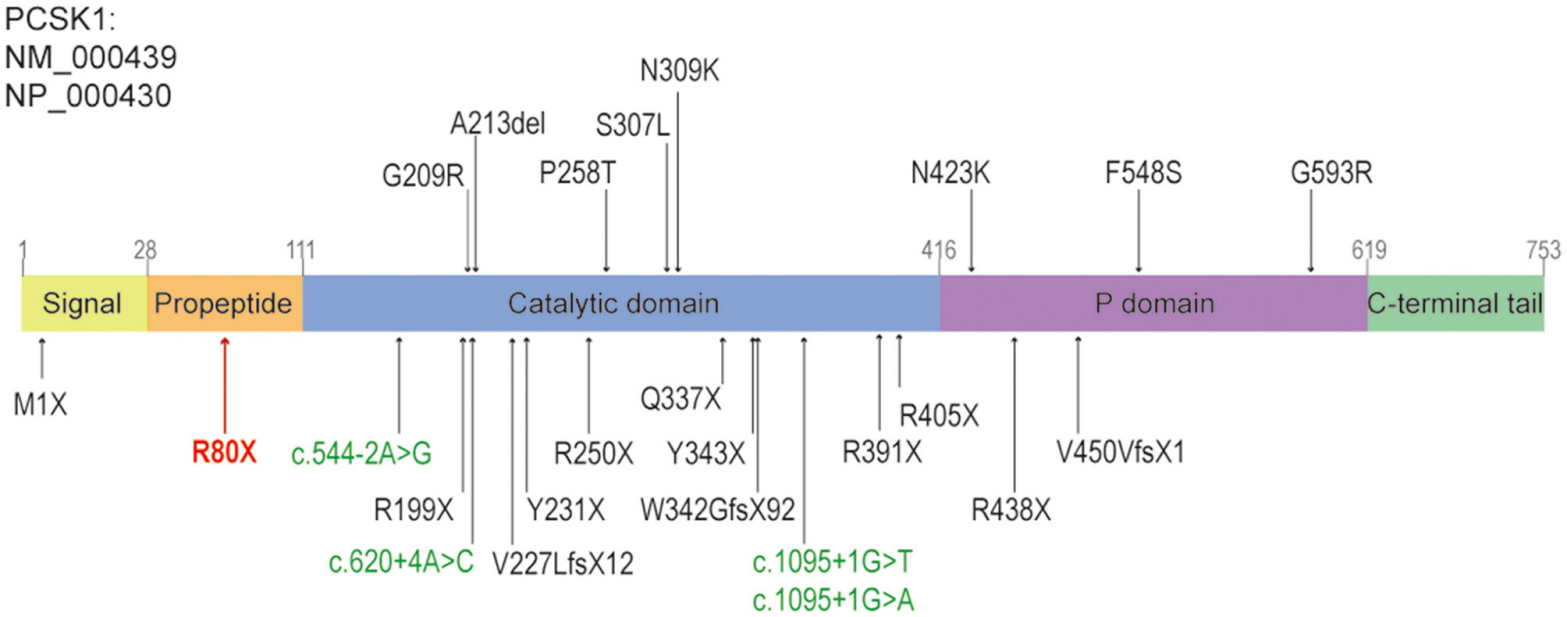
The structure of PCSK1, and the reported variants caused diarrhea, including this study. The missense variants and non-frameshift variants were shown above the panel, the nonsense, frameshift variants or splice sites were shown below the panel. The splice sites were shown in green, the nonsense variant identified in this study was shown in red. Revised from reference (Martin et al., 2013).

We updated the summary of patients with PC1/3 deficiency carrying PCSK1 variants from Lucie Pe'pin's review (Pepin et al., 2019). The first patient carrying compound heterozygous variants of *PCSK1* gene showing postnatal malabsorptive diarrhea and early-onset obesity was reported in 1997 (Jackson et al., 1997). Since then, 26 more cases of PC1/3 deficiency have been reported. Including our study, the 28 cases shared 25 distinct *PCSK1* gene variants (Pepin et al., 2019; Serra et al., 2020) (Figure 3). All of the 28 patients had early-onset severe malabsorptive diarrhea, and one of them was firstly diagnosed with inflammatory bowel disease (Serra et al., 2020). Among them, 96.3 percent (*n* = 26/27) are younger than 1 month (seven patients under 3 days old). Early-onset obesity (before 5 years) was observed in 80% (*n* = 20/25). Most of the patients got the genetic diagnosis when they suffered severe obesity and diabetes, or central diabetes insipidus. Our case was diagnosed at 8 months old. She was continually followed-up until 11 months; she could drink milk/water >1000 ml/day. She has not presented obesity or diabetes, but her BMI was increasing. So, diet control was suggested to prevent obesity and to control glucose levels.

From a molecular diagnose viewpoint, 72 and 20 percent of *PCSK1* pathogenic variants were located in the region coding for the catalytic domain and P domain, respectively. Sixty-eight percent of variants were deleterious, including 10 nonsense variants, 3 frameshift variants and 4 splice sites variants (Figure 3). However, there was no apparent genotype-phenotype correlation between individuals (Pepin et al., 2019).

As reported, 88.5 percent (23 of 26) families had a history of consanguinity (Martin et al., 2013; Pepin et al., 2019). Among the homozygous pathogenic variants, only one patient (Asn423Lys) was not in consanguinity but inherited from both parents (Martin et al., 2013). In this study, our patient had the homozygous variant inherited from the heterozygous carrier mother. Both array-CGH and Trio-ES data exclude any genomic deletion in this infant. Using the method to detect UPDs from Trio-ES data in our team (Zhang et al., 2020, 2021), we found that variants of the infant on chromosome 5 were 97.9% homozygous and maternally inherited, suggesting it was iUPD(5)mat. It is worth mentioning that there are three imprinting genes- *RHOBTB3, ERAP2*, and *VTRNA2-1* on chromosome 5. All of these regions were paternally imprinted, suggesting that imprinting is not a pathogenic mechanism for our patient. Till now, the reports of uniparental disomy of chromosome 5 due to homozygous mutant expression of recessive diseases are still rare. Our patient was the tenth case of UPD(5), and the fourth of UPD(5)mat. Different from the typical autosomal recessive disease, the recurrence risk for this family is relatively low.

In conclusion, we report the first case of PC1/3 deficiency caused by iUPD(5)mat with a nonsense homozygous variant of the *PCSK1* gene. Our results highlight that patients, particularly in those families without consanguinity, have the possibility of UPD as a cause of autosomal recessive disorders. The early genetic diagnosis for this girl may avoid unnecessary tests and provided precision treatment in time.

## Data Availability Statement

The datasets presented in this study can be found in online repositories. The names of the repository/repositories and accession number(s) can be found at: https://www.ncbi.nlm.nih.gov/, SUB8931511.

## Ethics Statement

The studies involving human participants were reviewed and approved by CHFU Ethics Committee. Written informed consent to participate in this study was provided by the participants' legal guardian/next of kin. Written informed consent was obtained from the minor(s)' legal guardian/next of kin for the publication of any potentially identifiable images or data included in this article.

## Author Contributions

HW, YH, CS, and BW conceived and supervised the project. RL, YL, and PZ contributed to data acquisition and analysis. YQ and HW wrote the manuscript. All authors approved the manuscript.

## Conflict of Interest

The authors declare that the research was conducted in the absence of any commercial or financial relationships that could be construed as a potential conflict of interest.
